# Hydrazones of 4-(Trifluoromethyl)benzohydrazide as New Inhibitors of Acetyl- and Butyrylcholinesterase

**DOI:** 10.3390/molecules26040989

**Published:** 2021-02-13

**Authors:** Martin Krátký, Katarína Svrčková, Quynh Anh Vu, Šárka Štěpánková, Jarmila Vinšová

**Affiliations:** 1 Department of Organic and Bioorganic Chemistry, Faculty of Pharmacy in Hradec Králové, Charles University, Akademika Heyrovského 1203, 500 05 Hradec Králové, Czech Republic; vuq@faf.cuni.cz (Q.A.V.); vinsova@faf.cuni.cz (J.V.); 2 Department of Biological and Biochemical Sciences, Faculty of Chemical Technology, University of Pardubice, Studentská 573, 532 10 Pardubice, Czech Republic; katarina.svrckova@upce.cz (K.S.); sarka.stepankova@upce.cz (Š.Š.)

**Keywords:** acetylcholinesterase inhibition, butyrylcholinesterase inhibition, enzyme inhibition, hydrazides, hydrazones, 4-(trifluoromethyl)benzohydrazide

## Abstract

Based on the broad spectrum of biological activity of hydrazide–hydrazones, trifluoromethyl compounds, and clinical usage of cholinesterase inhibitors, we investigated hydrazones obtained from 4-(trifluoromethyl)benzohydrazide and various benzaldehydes or aliphatic ketones as potential inhibitors of acetylcholinesterase (AChE) and butyrylcholinesterase (BuChE). They were evaluated using Ellman’s spectrophotometric method. The hydrazide–hydrazones produced a dual inhibition of both cholinesterase enzymes with IC_50_ values of 46.8–137.7 µM and 19.1–881.1 µM for AChE and BuChE, respectively. The majority of the compounds were stronger inhibitors of AChE; four of them (2-bromobenzaldehyde, 3-(trifluoromethyl)benzaldehyde, cyclohexanone, and camphor-based **2o**, **2p**, **3c**, and **3d**, respectively) produced a balanced inhibition of the enzymes and only 2-chloro/trifluoromethyl benzylidene derivatives **2d** and **2q** were found to be more potent inhibitors of BuChE. 4-(Trifluoromethyl)-*N*’-[4-(trifluoromethyl)benzylidene]benzohydrazide **2l** produced the strongest inhibition of AChE via mixed-type inhibition determined experimentally. Structure–activity relationships were identified. The compounds fit physicochemical space for targeting central nervous systems with no apparent cytotoxicity for eukaryotic cell line together. The study provides new insights into this CF_3_-hydrazide–hydrazone scaffold.

## 1. Introduction

An increasing interest in fluorinated compounds is due to the favorable effect of fluorine on pharmacological properties. It can be conservatively estimated that globally about 20–25% of drugs in the pharmaceutical pipeline contain at least one fluorine atom [1]. Fluorine is making an impact in pharmaceuticals not only in a fast-growing number of fluorinated drugs but also in the development of many of the best health care products [2]. The special properties that make fluorinated motifs very attractive in medicinal chemistry and chemical biology include small atomic radius, high electronegativity, nuclear spin of one-half, and low polarizability of the C–F bond [3,4]. Substituting C–H by C–F bonds clearly benefits from the fact that the size of organofluorine is only slightly larger than the size and volume of the hydrogen substituent and that consequently no particular steric hindrance is encountered in most H→F replacements [5]. Among the fluorinated groups such as F, CF_3_, OCF_3_, CHF_2_ etc., CF_3_ is a very familiar pharmacophore [6]. This abiotically synthesized group—usually replacing chlorine, methyl, ethyl, or isopropyl group—is a frequently used bioisostere in medicinal chemistry, dating from 1929 [7]. This electron withdrawing functionality increases the biological half-life by impeding the oxidative metabolism, and its lipophilicity improves cellular membrane permeability, thus increasing absorption [8,9].

**Figure 1:**
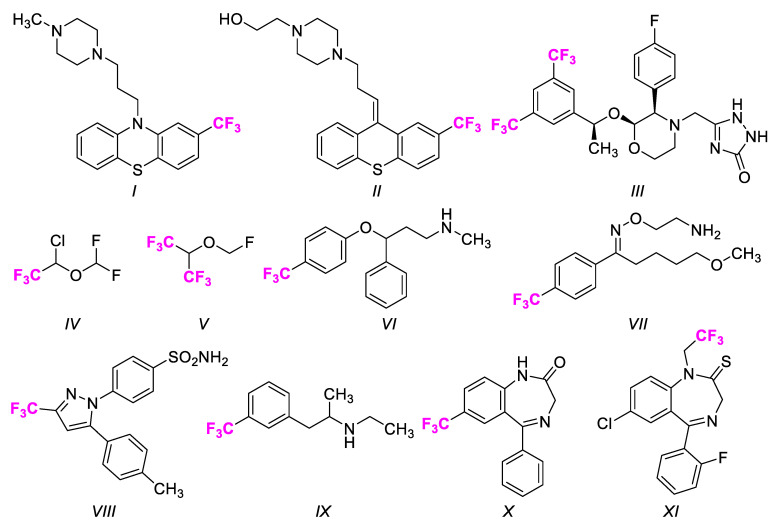
Examples of trifluoromethyl motif containing CNS-acting drugs.

Given together, the CF_3_ group belongs to common and useful motifs in central nervous system (CNS)-acting drugs—for example, antipsychotics trifluoperazine (I) and flupentixol (II), aprepitant (III) used against nausea and vomitus; central anesthetics isoflurane (IV) and sevoflurane (V); fluoxetine (VI) and fluvoxamine (VII) prescribed as antidepressants; centrally acting nonsteroidal anti-inflammatory drug celecoxib (VIII); anorectic drug fenfluramine (IX); and benzodiazepine anxiolytics, hypnotics, and anticonvulsants such as triflunordazepam (X) and quazepam (XI) (Figure 1).

Compounds bearing hydrazide–hydrazone scaffold (−CO–NH–N═C(R)−; i.e., fused hydrazide and hydrazone moieties) have a substantial beneficial value in the development of novel antibacterial, mainly antitubercular, antiplatelet, anticancer, anti-inflammatory, and, with regard to targeting the CNS, also of anticonvulsant, analgesic, and antidepressant agents [10,11].

4-(Trifluoromethyl)benzohydrazide (**1**)-based hydrazones have been reported as potential antimicrobial agents active against Gram-positive and Gram-negative bacteria, mycobacteria, yeasts, and molds [12,13,14]. Moreover, hydrazones of **1** have exhibited inhibition of various enzymes, i.e., bacterial DNA-gyrase and FabH (β-ketoacyl-acyl carrier protein synthase III) [15], urease [14], cyanobacterial fructose-1,6-/sedoheptulose-1,7-bisphosphatase [16] and, more importantly, also acetylcholinesterase (AChE) and butyrylcholinesterase (BuChE) [17,18]. The enzyme inhibiting properties are not unique for the CF_3_ group bearing hydrazide–hydrazones, of course. Illustratively, hydrazones derived from 4-hydroxybenzohydrazide have been studied as a new class of dual inhibitors of thymidine phosphorylase and cancer cell proliferation, which deserves to be further investigated for anti-cancer drug development [19], as well as indole-based acetohydrazide inhibitors of this enzyme [20]. Also, xanthinoxidase can be inhibited by hydrazide–hydrazones in free form as well as after their coordination to copper (II) [21]. Laccase is a copper-containing polyphenol oxidase that catalyzes the radical reduction of atmospheric oxygen to water with simultaneous oxidation of electron-rich aromatic compounds (polyphenols, anilines). Inhibition of this enzyme might weaken activity of the plant pathogens that utilize it in various biochemical processes. The relationship between 4-hydroxybenzohydrazide-hydrazones and laccase from *Trametes versicolor* was studied and a competitive type of inhibition was identified [22]. In our previous study [23], *N*-alkyl-2-[4-(trifluoromethyl)benzoyl]hydrazine-1-carboxamides were identified as inhibitors of both AChE and BuChE. Based on here presented findings, we investigated our trifluoromethylated hydrazide–hydrazones **2**–**3** (Table 1) [12] as potential inhibitors of AChE a BuChE together with their newly prepared analogues **2n**–**2u**.

## 2. Results and Discussion

### 2.1. Chemistry

**Scheme 1:**
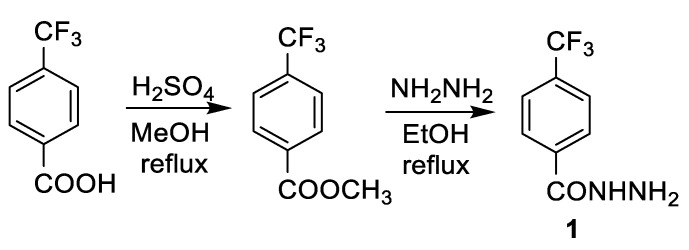
Synthesis of the 4-(trifluoromethyl)benzohydrazide **1**.

**Scheme 2:**
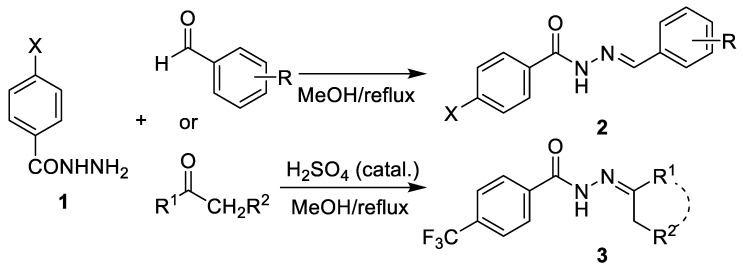
Synthesis of the hydrazide–hydrazones **2** and **3** [X = CF_3_ (**2a**-**2s**), H (**2t**), CH_3_ (**2u**)].

The title hydrazide **1** was prepared from 4-(trifluoromethyl)benzoic acid by a two-step process. First, this acid was esterified by methanol in the presence of a catalytic amount of sulfuric acid under heating in an excess of the alcohol. Then, the methyl ester was converted to hydrazide by hydrazinolysis using hydrazine hydrate in boiling ethanol (Scheme 1). The overall yield was almost quantitative. This procedure was described previously by our group for 4-iodobenzohydrazide [24]. The hydrazone–hydrazides were synthesized by the treatment of **1** (1 mmol) with various aldehydes and ketones (1.1 mmol) in boiling methanol for 2 h (Scheme 2); for ketones with acidic catalyst (concentrated sulfuric acid). The yields of the hydrazide–hydrazones **2** and **3** ranged from 85% to 99% and from 68% to 87%, respectively. The characterization of the compounds **2a**–**2m** and **3** was reported in depth by Krátký et al. [12]; the remaining ones were prepared newly on the basis of the results of biological evaluation for a deeper insight into structure–activity relationships. The group of novel compounds covers positional isomers of the most effective AChE inhibitors (**2k**–**2m**), i.e., the derivatives **2n**–**2s**, as well as hydrazide–hydrazones without 4-CF_3_-benzohydrazide moiety (trifluoromethyl was removed, **2t**, or replaced by methyl, **2u**).

### 2.2. Inhibition of Acetyl- and Butyrylcholinesterase

**Figure 2:**
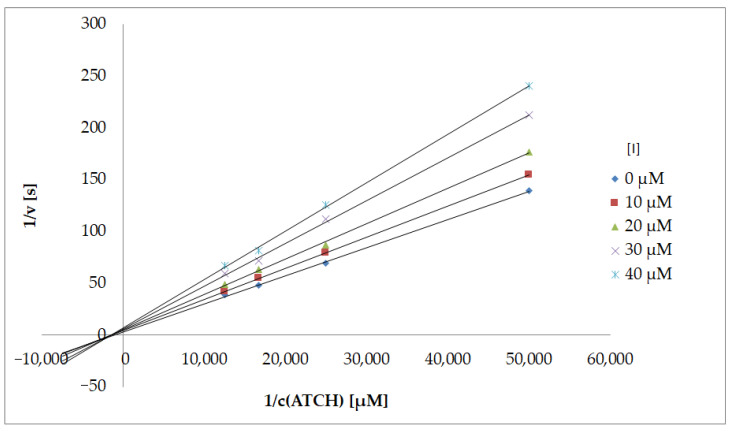
Lineweaver–Burk plot for **2l** inhibiting eeAChE (ATCh = acetylthiocholine; [I] = concentration of inhibitor).

The hydrazide–hydrazones **2** and **3** as well as the parent compound **1** were screened for their potential to affect the function of AChE isolated from electric eel (*Ee*AChE) and BuChE obtained from equine serum (EqBuChE) using Ellman’s spectrophotometric method (Table 1). These enzymes of nonhuman origin were used to screen inhibitory activity and to compare the results with our previous compounds sharing a certain degree of a chemical similarity [23,24]. Their efficacy is expressed as the concentration leading to 50% inhibition of the enzymatic activity (IC_50_). To quantify the preference to particular acetylcholine decomposing enzymes, selectivity indexes (SI) were calculated. SI is defined as a ratio of IC_50_ for BuChE/IC_50_ for AChE (Table 1; illustrated also in Figures S1–S6). Values higher than 1 mean stronger inhibition of AChE and vice versa. Based on analogy with therapeutic index, SI over the borderline of 10 indicates selective AChE inhibition. In addition to **1**, galantamine was employed as a standard for comparison of IC_50_ values. Moreover, we investigated the type of inhibition of the most active AChE inhibitor identified (**2l**; Figure 2, Table S1).

The original [12] hydrazide–hydrazones **2a**–**2m** and **3** exhibited a dual inhibition of both enzymes with IC_50_ values of 46.8–137.7 µM and 63.6–881.1 µM for AChE and BuChE, respectively. Focusing on the benzaldehyde derivatives **2** and AChE, 4-(trifluoromethyl)benzylidene hydrazone **2l** was the most in vitro efficient, together with salicylidene hydrazone **2g**. The introduction of unsubstituted benzylidene moiety into the molecule **1** resulted in a comparable activity (**1** vs. **2a**). The inhibition potency was improved when an electronegative substituent was present at the position 4: second CF_3_ group (–I effect; **2l**), nitro group (–I and –M effects; **2m**), or bromine (+M and predominant –I effect; **2k**). The replacement of bromine by chlorine led to a mild drop in the activity (**2k** vs. **2b**). On the other hand, electron-donating substituents were unfavorable (methyl **2i**, methoxy **2j**). We also evaluated chlorine and hydroxyl group positional isomers (**2b**–**2d** and **2e**–**2g**, respectively); however, there was no clear structure–activity relationship. Interestingly, the combination of these two groups at their best positions led to the less potent compound within this study (**2h**).

IC_50_ values of **2** for BuChE were clearly higher. 2-Chlorobenzylidene analogue **2d** showed the lowest value, followed by approximately 3-fold less active 4-CF_3_ (**2l**) and 4-NO_2_ (**2k**) substituted hydrazones. Contrarily, benzylidene (**2a**), salicylidene (**2g**), and 4-bromobenzylidene (**2k**) substituents were detrimental to inhibition. Remaining derivatives produced similar IC_50_ values. The positional isomers of **2d** shared significantly lower inhibitory potency.

Among ketone-derived hydrazones **3**, the inhibition of AChE was virtually uniform (59–70 µM); bigger ketones (camphor, cyclohexanone) were preferred over smaller ones (acetone, cyclopentanone). This structure–activity dependence was even more obvious in the case of BuChE inhibition (**3a**→**3d**, from 424 to 57 µM), favoring again cyclohexanone and especially camphor as starting scaffolds.

Then, we also evaluated additional derivatives **2n**–**2u**. The positional isomers of the brominated derivative **2k** provided an improved activity against BuChE for both ortho- and meta-isomers (up to 6.3×, **2k** vs. **2o**), although meta-isomer **2n** was less effective against AChE. Analogous structure–activity relationships were also found for the novel nitro derivatives **2r** and **2s**. The hydrazones based on 2- and 3-CF_3_-benzaldehyde (**2p** and **2q**) retained activity against AChE but were significantly more effective against BuChE, especially the ortho-isomer **2q** with IC_50_ of 19.1 µM, i.e., the most active BuChE inhibitor in this study. In sum, especially 2-substituted benzaldehydes-based hydrazones were beneficial when compared to their original para-isomers in terms of improved activity against BuChE along with the same inhibition of AChE. Meta-isomers also led to a more balanced inhibition of both acetylcholine hydrolyzing enzymes.

Focusing on the removal of CF_3_ group (**2t**) and its replacement by CH_3_ group (**2u**), these modifications led to a decreased activity, although these two derivatives were also capable of inhibiting both AChE and BuChE. The benzohydrazide derivative **2t** was a more potent inhibitor.

The modification of the parent hydrazide **1** led to 11 derivatives that were more active against AChE (**2g**, **2k**–**2m**, **2o**–**2q**, **2s**, **2t**, **3c**, and **3d**) and 5 others were fully comparable (**2a**, **2f**, **2u**, **3a**, and **3b**). For BuChE, 13 compounds (**2d**, **2l**, **2m**–**2t**, **3b**–**3d**) exhibited lower IC_50_ than the hydrazide **1**. Then, we compared the results with those obtained for galantamine, the highly active inhibitor of AChE approved for the treatment of Alzheimer’s disease-related dementia. However, none of the hydrazide–hydrazones exhibited comparable IC_50_ values.

Focusing on selectivity toward cholinesterases, the majority of the hydrazide–hydrazones **2**–**3** inhibited AChE more strongly (selectivity indexes, SI, ≥2). 2-Hydroxybenzylidene derivative **2g**, the second most potent AChE inhibitor, was the most selective AChE inhibitor (SI = 18.6), followed by unsubstituted benzylidene **2a** (8.7), propan-2-ylidene **3a** (6.1), and 4-bromobenzylidene analogue **2k** (SI of 5.5). On the other hand, only the presence of 2-chlorobenzylidene and 2-(trifluoromethyl)benzylidene substituents (**2d** and **2q**) led to a higher inhibition of BuChE; however, not selectively (SI = 0.5 and 0.4, respectively). Two larger ketone-based hydrazones obtained from cyclohexanone (**3c**) and camphor (**3d**) produced a balanced inhibition of both acetylcholine hydrolyzing enzymes as well as the remaining hydrazones of isomeric 2-/3-bromo- and nitrobenzaldehydes (**2n**, **2o**, **2r**, and **2s**) and 3-CF_3_-benzaldehyde (**2p**).

Moreover, all the original derivatives were without any signs of cytotoxicity for HepG2 cells at 100 µM (Table 1) [12].

#### Type of Inhibition

The reversible enzyme inhibitors can be classified into four groups: competitive, non-competitive, uncompetitive, and mixed type of inhibition. The type of inhibition could be distinguished using a Lineweaver–Burk plot [25] and corresponding comparison of two kinetic parameters: Michaelis constant (K_M_) and maximum velocity (V_m_) of uninhibited and inhibited reaction. The Lineweaver–Burk plot of the most potent AChE inhibition with **2l** is depicted in Figure 2. Based on the plot of K_M_ and V_m_, this hydrazide–hydrazone caused a mixed type of inhibition.

### 2.3. Prediction of BBB Permeability

In addition to their efficacy and low toxicity, compounds intended for treatment of dementias must be able to cross the blood-brain barrier (BBB) to reach their target site. To predict this crucial issue in silico, various empirical approaches have been proposed and validated. They are based on various physicochemical and structural parameters inferred from known CNS drugs.

Classical selection criteria include molecule size, lipophilicity, number of hydrogen bond donors and acceptors, aromatic rings, basic groups, heteroatoms, polar surface area, molar refractivity, molecular flexibility quantified by a number of rotatable single bonds, p*K*_a_, etc. [26,27,28,29]. In general, lipophilicity is regarded to be the most important property, and its increased value often results in an improved in vitro activity [27].

The data (M_w_, consensus log *P*, number of hydrogen bond donors and acceptors, rotatable bonds, and topological polar surface area (tPSA)) of the derivatives **1**–**3** (Table 2) were compared with margins proposed as ideal for CNS drugs [29]. The vast majority of these parameters were within the suggested ranges; only several compounds (**2j**, **2l**, **2m**, and **2p**–**2s**) had one more rotatable bond. Nevertheless, delivery across the BBB is not bounded sharply. Since it is a consequence of many factors, the cut-offs are not strict, and they are related to a higher probability of targeting the CNS.

In addition to this structural multiparameter appraisal, we also involved a newer BOILED-Egg (Brain Or IntestinaL EstimateD permeation) approach [26] based on lipophilicity and polarity of small molecules to predict penetration through the BBB via passive diffusion. Passive diffusion usually represents a key entrance route for centrally acting drugs, and it requires a certain degree of lipophilicity (log *P*) in combination with a mild polarity (tPSA). To estimate brain permeability using BOILED-Egg, the SwissADME [30] calculator was utilized.

The results are summarized in Table 2. With the exception of 4-CF_3_ derivative **2l** and their isomers **2p** and **2q** (due to enhanced lipophilicity) and 4-NO_2_ compounds (**2m**, **2r**, and **2s**), the remaining hydrazide–hydrazones met the requirements for CNS active molecules. However, the BOILED-Egg approach-based prediction often failed in the prediction of BBB permeability of nitro group-containing drugs that are indicated far from the optimal physicochemical space, even in the case of typical CNS drugs and drugs exhibiting their pharmacologic effects in the brain (e.g., calcium channel blockers including nitrendipine, nilvadipine and nimodipine, non-steroidal anti-inflammatory agent nimesulide, tolcapone used as a levodopa booster, organophosphorus AChE inhibitors such as paraoxon and parathion, benzodiazepine hypnotics nitrazepam and flunitrazepam, antibiotic chloramphenicol, etc.). Thus, the prediction seems to be unreliable for nitro derivatives.

Furthermore, BOILED-Egg was useful for prediction of gastrointestinal absorption after oral administration based on the identical physicochemical descriptors. Hopefully, all the derivatives **1**–**3** meet in silico criteria for high gastrointestinal absorption; only for bis-CF_3_ analogues it was predicted to be lower.

## 3. Materials and Methods

### 3.1. Chemistry

General. All the reagents and solvents were purchased from Sigma-Aldrich (Darmstadt, Germany) and Penta Chemicals (Prague, Czech Republic), and they were used as received. The progress of the reactions and the purity of the products were monitored by thin layer chromatography using a mixture of toluene with ethyl acetate and tetrahydrofuran (4:1:0.3, *v*/*v*) as an eluent; plates were coated with 0.2 mm Merck 60 F254 silica gel (Merck Millipore, Darmstadt, Germany) and were visualized by UV irradiation (254 nm). Melting points were determined on a Büchi Melting Point B-540 apparatus (BÜCHI, Flawil, Switzerland) using open capillaries; the reported values are uncorrected.

Elemental analysis (C, H, N) was performed on a Vario MICRO Cube Element Analyzer (Elementar Analysensysteme, Hanau, Germany). Both calculated and found values are given as percentages. Infrared spectra (ATR) were recorded on a Nicolet 6700 FT-IR spectrometer (ThermoFisher Scientific, Waltham, MA, USA) in the range of 600 to 4000 cm^−1^. The NMR spectra were measured in DMSO-*d*_6_ at ambient temperature on a Varian V NMR S500 instrument (500 MHz for ^1^H and 125 MHz for ^13^C; Varian Comp., Palo Alto, CA, USA). The chemical shifts, δ, are given in ppm with respect to tetramethylsilane via signals of DMSO-*d*_6_ (2.49 for ^1^H and 39.7 for ^13^C spectra). The coupling constants (*J*) are reported in Hz.

The synthesis and characterization of the compounds **2a**–**2m** and **3** were reported in depth by Krátký et al. [12].

The novel derivatives were prepared and purified according to an identical procedure. Briefly, an appropriate hydrazide (1 mmol) was dissolved in 6 mL of MeOH and then a substituted benzaldehyde was added in one portion (1.1 of equivalents) under vigorous stirring. The reaction mixture was stirred at rt for 5 min and then refluxed for 2 h. Resulting suspension was let to stay overnight at +4 °C and then was filtered off. The resulting crystals were washed with a small volume of cold MeOH and diethyl ether and then dried to obtain pure hydrazones **2n**–**2u**.

*N*’-(3-Bromobenzylidene)-4-(trifluoromethyl)benzohydrazide **2n**. White solid; yield 96%; mp 193–195 °C. IR (ATR): 3018, 1654, 1558, 1327, 1304, 1290, 1164, 1151, 1118, 1108, 1067, 1019, 962, 927, 853, 786, 770, 683 cm^−1^. ^1^H-NMR (500 MHz, DMSO-*d*_6_): δ 12.19 (1H, bs, NH), 8.47 (1H, s, CH=N), 8.12 (2H, d, *J* = 8.3 Hz, H2, H6), 7.96–7.90 (3H, m, H3, H5, H2’), 7.75 (1H, dt, *J* = 7.8, 1.3 Hz, H6´), 7.64 (1H, dt, *J* = 8.0, 1.5 Hz, H4´), 7.43 (1H, t, *J* = 7.8 Hz, H5´). ^13^C-NMR (126 MHz, DMSO): δ 162.58, 147.23, 137.49, 137.07, 133.25, 132.09 (q, *J* = 32.0 Hz), 131.50, 129.73, 129.09, 126.79, 125.96 (q, *J* = 3.8 Hz), 124.33 (q, *J* = 272.8 Hz), 122.66. Analytically calculated for C_15_H_10_BrF_3_N_2_O (371.15): C, 48.54; H, 2.72; N, 7.44. Found: C, 48.52; H, 2.77; N, 7.40.

*N*’-(2-Bromobenzylidene)-4-(trifluoromethyl)benzohydrazide **2o**. White solid; yield 97%; mp 217–219.5 °C. ^1^H-NMR (500 MHz, DMSO-*d*_6_): δ 12.30 (1H, bs, NH), 8.84 (1H, s, CH=N), 8.15 (2H, d, *J* = 8.1 Hz, H2, H6), 8.02 (1H, dd, *J* = 7.9, 1.8 Hz, H6´), 7.92 (2H, d, *J* = 8.2 Hz, H3, H5), 7.70 (1H, dd, *J* = 8.0, 1.3 Hz, H3´), 7.50–7.45 (1H, m, H5´), 7.38 (1H, td, *J* = 7.6, 1.8 Hz, H4´). ^13^C-NMR (126 MHz, DMSO): δ 162.49, 147.27, 137.40, 133.67, 133.36, 132.41, 132.14 (q, *J* = 32.0 Hz), 129.11, 128.62, 127.79, 125.97 (q, *J* = 3.8 Hz), 124.33 (q, *J* = 272.3 Hz), 124.18. Analytically calculated for C_15_H_10_BrF_3_N_2_O (371.15): C, 48.54; H, 2.72; N, 7.44. Found: C, 48.50; H, 2.78; N, 7.48.

4-(Trifluoromethyl)-*N*’-[3-(trifluoromethyl)benzylidene]benzohydrazide **2p**. White solid; yield 90%; mp 162.5–164.5 °C. IR (ATR): 3007, 1649, 1570, 1327, 1306, 1292, 1281, 1217, 1167, 1126, 1111, 1068, 1062, 1019, 945, 854, 803, 770, 698, 670 cm^−1^. ^1^H-NMR (500 MHz, DMSO-*d*_6_): δ 12.27 (1H, bs, NH), 8.55 (1H, s, CH=N), 8.13 (2H, d, *J* = 8.1 Hz, H2, H6), 8.09–8.03 (2H, m, H2´, H6´), 7.92 (2H, d, *J* = 8.1 Hz, H3, H5), 7.80 (1H, d, *J* = 7.9 Hz, H4´), 7.71 (1H, t, *J* = 7.8 Hz, H5´). ^13^C-NMR (126 MHz, DMSO): δ 162.43, 147.00, 137.20, 135.54, 131.87 (q, *J* = 31.9 Hz), 131.38, 130.29, 129.89 (q, *J* = 31.9 Hz), 128.86, 126.72 (q, *J* = 3.8 Hz), 125.73 (q, *J* = 3.9 Hz), 124.22 (q, *J* = 272.3 Hz), 124.07 (q, *J* = 272.8 Hz), 123.35 (q, *J* = 3.9 Hz). Analytically calculated for C_16_H_10_F_6_N_2_O (360.25): C, 53.34; H, 2.80; N, 7.78. Found: C, 53.32; H, 2.83; N, 7.90.

4-(Trifluoromethyl)-*N*’-[2-(trifluoromethyl)benzylidene]benzohydrazide **2q**. White solid; yield 95%; mp 182.5–184 °C. IR (ATR): 3034, 1651, 1559, 1489, 1316, 1290, 1278, 1170, 1160, 1137, 1112, 1104, 1062,1032, 1020, 964, 860, 766, 671 cm^−1^. ^1^H-NMR (500 MHz, DMSO-*d*_6_): δ 12.36 (1H, bs, NH), 8.85 (1H, q, *J* = 2.2 Hz, CH=N), 8.25 (1H, d, *J* = 7.9 Hz, H6´), 8.14 (2H, d, *J* = 8.1 Hz, H2, H6), 7.93 (2H, d, *J* = 8.2 Hz, H3, H5), 7.83–7.76 (2H, m, H3´, H5´), 7.65 (1H, t, *J* = 7.7 Hz, H4´). ^13^C-NMR (126 MHz, DMSO): δ 162.62, 144.00, 137.29, 133.37, 132.46, 132.21 (q, *J* = 31.9 Hz), 130.77, 129.13, 127.40, 127.37 (q, *J* = 32.0 Hz), 126.42 (q, *J* = 3.9 Hz), 125.98 (q, *J* = 3.7 Hz), 124.62 (q, *J* = 272.7 Hz), 124.32 (q, *J* = 272.3 Hz). Analytically calculated for C_16_H_10_F_6_N_2_O (360.25): C, 53.34; H, 2.80; N, 7.78. Found: C, 53.39; H, 2.77; N, 7.72.

*N*’-(3-Nitrobenzylidene)-4-(trifluoromethyl)benzohydrazide **2r**. Yellowish solid; yield 99%; mp 205–207 °C. IR (ATR): 3205, 3055, 1665, 1551, 1527, 1343, 1327, 1282, 1179, 1149, 1132, 1113, 1066, 1019, 949, 936, 898, 862, 814, 771, 738, 690, 669 cm^−1^. ^1^H-NMR (500 MHz, DMSO-*d*_6_): δ 12.31 (1H, bs, NH), 8.58–8.55 (2H, m, CH=N, H2´), 8.28–8.25 (1H, m, H4´), 8.17 (1H, dd, *J* = 7.8, 1.4 Hz, H6´), 8.13 (2H, d, *J* = 8.0 Hz, H2, H6), 7.92 (2H, d, *J* = 8.1 Hz, H3, H5), 7.76 (1H, t, *J* = 8.0 Hz, H5´). ^13^C-NMR (126 MHz, DMSO): δ 162.70, 148.68, 146.59, 137.36, 136.46, 133.95, 132.15 (q, *J* = 31.7 Hz), 130.95, 129.13, 125.98 (q, *J* = 3.9 Hz), 124.90, 124.32 (q, *J* = 272.4 Hz), 121.50. Analytically calculated for C_15_H_10_F_3_N_3_O_3_ (337.25): C, 52.42; H, 2.99; N, 12.46. Found: C, 52.43; H, 2.95; N, 12.53.

*N*’-(2-Nitrobenzylidene)-4-(trifluoromethyl)benzohydrazide **2s**. Yellowish solid; yield 98%; mp 215.5–216.5 °C. IR (ATR): 3201, 3089, 1651, 1554, 1522, 1365, 1343, 1323, 1292, 1175, 1152, 1131, 1113, 1065, 1018, 955, 922, 855, 788, 744, 702, 687 cm^−1^. ^1^H-NMR (500 MHz, DMSO-*d*_6_): δ 12.35 (1H, bs, NH), 8.86 (1H, s, CH=N), 8.13–8.04 (4H, m, H3, H5, H3´, H6´), 7.89 (2H, d, *J* = 8.1 Hz, H2, H6), 7.79 (1H, d, *J* = 7.6 Hz, H5´), 7.66 (1H, t, *J* = 7.8 Hz, H4´). ^13^C-NMR (126 MHz, DMSO): δ 162.73, 148.84, 144.44, 137.33, 134.32, 132.29 (q, *J* = 32.2 Hz), 131.41, 129.29, 129.13, 128.51, 126.06 (q, *J* = 3.8 Hz), 125.30, 124.43 (q, *J* = 272.3 Hz). Analytically calculated for C_15_H_10_F_3_N_3_O_3_ (337.25): C, 52.42; H, 2.99; N, 12.46. Found: C, 52.37; H, 3.06; N, 12.55.

*N*’-[4-(Trifluoromethyl)benzylidene]benzohydrazide **2t**. White solid; yield 99%; mp 188–189 °C. IR (ATR): 3290, 1664, 1548, 1377, 1325, 1305, 1281, 1171, 1156, 1147, 1107, 1067, 1018, 953, 856, 839, 718, 692, 670 cm^−1^. ^1^H-NMR (500 MHz, DMSO-*d*_6_): δ 12.06 (1H, bs, NH), 8.54 (1H, s, CH=N), 7.98–7.91 (4H, m, H2, H6, H2´, H6´), 7.82 (2H, d, *J* = 8.0 Hz, H3´, H5´), 7.61 (1H, d, *J* = 7.3 Hz, H4), 7.54 (2H, t, *J* = 7.5 Hz, H3, H5). ^13^C-NMR (126 MHz, DMSO): δ 163.81, 146.44, 138.79, 133.66, 132.41, 130.15 (q, *J* = 31.6 Hz), 128.99, 128.16, 128.12, 126.20 (q, *J* = 3.9 Hz), 124.57 (q, *J* = 272.2 Hz). Analytically calculated for C_15_H_11_F_3_N_2_O (292.26): C, 61.65; H, 3.79; N, 9.59. Found: C, 61.60; H, 3.85; N, 9.63.

4-Methyl-*N*’-[4-(trifluoromethyl)benzylidene]benzohydrazide **2u**. White solid; yield 94%; mp 212–213.5 °C. IR (ATR): 3277, 1661, 1545, 1386, 1369, 1323, 1280, 1159, 1146, 1120, 1105, 1066, 1017, 950, 838, 750, 672 cm^−1^. ^1^H-NMR (500 MHz, DMSO-*d*_6_): δ 11.93 (1H, bs, NH), 8.49 (1H, s, CH=N), 7.90 (2H, d, *J* = 8.0 Hz, H2, H6), 7.83–7.76 (4H, m, H2´, H3´, H5´, H6´), 7.30 (2H, d, *J* = 7.9 Hz, H3, H5), 2.34 (3H, s, CH_3_). ^13^C-NMR (126 MHz, DMSO): δ 163.69, 146.32, 142.57, 138.95, 130.85, 130.16 (q, *J* = 31.5 Hz), 129.58, 128.26, 128.15, 126.26 (q, *J* = 3.8 Hz), 124.66 (q, *J* = 272.2 Hz), 21.57. Analytically calculated for C_16_H_13_F_3_N_2_O (306.29): C, 62.74; H, 4.28; N, 9.15. Found: C, 62.69; H, 4.24; N, 9.12.

### 3.2. Evaluation of Acetyl- and Butyrylcholinesterase Inhibition

The activities of the investigated compounds **2**–**3** were measured via Ellman’s spectrophotometric method that was modified according to Zdražilová et al. [31] to determine the IC_50_ values. Ellman’s method has been widely applied for the screening of the efficiency of potential AChE and BuChE inhibitors. The activity of cholinesterases was assessed indirectly by quantifying the amount of 5-mercapto-2-nitrobenzoic acid ion formed in the reaction of the thiol reagent 5,5′-disulfanediylbis(2-nitrobenzoic acid) with thiocholine, a product of the hydrolysis of acetyl- (ATCh) or butyrylthiocholine (BTCh) catalyzed by AChE and BuChE, respectively.

AChE used in this assay was obtained from electric eel (*Electrophorus electricus* L.) and BuChE from equine serum. The benzazepine alkaloid drug galantamine was used as a reference enzyme inhibitor. All the enzymes, the thiol reagent, ATCh, BTCh, and galantamine were purchased from Sigma-Aldrich (Prague, Czech Republic).

The enzyme activity in final reaction mixture (2 mL) was 0.2 U/mL, the concentration of ATCh or BTCh was 40 µM, and the concentration of 5,5′-disulfanediylbis(2-nitrobenzoic acid) was 0.1 mM for all reactions. The investigated hydrazide–hydrazones were dissolved in DMSO and then diluted by demineralized water (conductivity 3 μS, equipment supplier BKG Water Treatment, Hradec Králové, Czech Republic) to the concentration of 1 mM. For all of them as well as galantamine, five different concentrations of inhibitor in final reaction mixture were used. All measurements were carried out in triplicates, and the average values of reaction rate (v_0_-uninhibited reaction, v_i_-inhibited reaction) were used for construction of the dependence v_0_/v_i_ vs. concentration of inhibitor. From the obtained equation of the regression curve, IC_50_ values were calculated.

For the determination of the type of inhibition of the most active AChE inhibitor **2l**, a Lineweaver–Burk plot [25] was used, and the measuring procedure was analogous to the determination of IC_50_. The enzyme activity in the final reaction mixture (2 mL) was 0.2 U/mL, a concentration of ATCh was 20–80 µM, and a concentration of 5,5′-disulfanediylbis(2-nitrobenzoic acid) was 0.1 mM. For each of the substrate concentrations, four different concentrations of the inhibitor were used (10, 20, 30, and 40 µM). The dependence absorbance vs. time was observed and the reaction rate was calculated. All measurements were carried out in duplicate and the average values of reaction rate were used for the construction of the Lineweaver–Burk plot. Furthermore, the dependencies vs. concentration of ATCh in absence and presence of inhibitor **2l** were analyzed by nonlinear regression using GraphPad Prism 9.0.1 for Windows (GraphPad Software, San Diego, CA, USA, www.graphpad.com). From obtained equations of regression curves in the Lineweaver–Burk plot, the values of Michaelis constant (K_M_) and maximum velocity (V_m_) were calculated. Using nonlinear regression (chosen panel: enzyme kinetics—inhibition/mixed model inhibition) the values of K_I_ (54.63 µM) and alpha (1.28) were determined. Based on all obtained results, the type of inhibition was identified.

### 3.3. Determination of Parameters for CNS Delivery

The parameters for in silico prediction of CNS delivery (log *P* as a value of consensus Log *P*_o/w_, number of rotatable bonds, and topological polar surface area, tPSA) as well as BOILED-Egg [26] method estimation of brain permeability were calculated using a free web tool SwissADME [30]; http://www.swissadme.ch/index.php.

## 4. Conclusions

We screened twenty-five hydrazide–hydrazones for their in vitro potency to inhibit AChE and BuChE. These compounds were derivatives of 4-(trifluoromethyl)benzohydrazide and various aromatic aldehydes and aliphatic ketones of choice. In addition, we evaluated the influence of various substitution patterns of benzaldehyde. IC_50_ values found were in the micro molar range from 46.8 µM for AChE and 19.1 µM for BuChE. Most of the derivatives were better inhibitors of AChE. 2-/3-/4-(Trifluoromethyl)-, 2-/4-nitro-, 2-/4-bromo-, and 2-hydroxybenzaldehydes together with camphor and cyclohexanone represented the most successful carbonyl compounds used for the modification of the parent hydrazide. Focusing on the type of inhibition, the most potent AChE inhibitor **2l** derived from 4-(trifluoromethyl)benzaldehyde exhibited a mixed inhibition of the enzyme. After in silico evaluation, the hydrazide–hydrazones shared generally convenient properties for CNS delivery and gastrointestinal absorption.

## Figures and Tables

**Table 1:** Inhibition of AChE and BuChE, selectivity and toxicity data of **1**–**3**.

	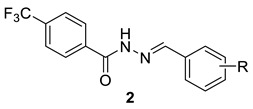				
Code	R	IC_50_ AChE (µM)	IC_50_ BuChE (µM)	Selectivity BuChE/AChE	IC_50_ (HepG2) [12]
**2a**	H	75.90 ± 1.6	651.3 ± 31.5	8.7	>100
**2b**	4-Cl	88.5 ± 1.4	253.8 ± 3.3	2.9	>100
**2c**	3-Cl	77.1 ± 0.03	260.9 ± 40.7	3.4	>100
**2d**	2-Cl	128.5 ± 10.0	**63.6 ± 0.9**	**0.5**	>100
**2e**	4-OH	101.9 ± 3.8	229.2 ± 14.5	2.3	>100
**2f**	3-OH	70.4 ± 2.8	249.7 ± 7.5	3.5	>100
**2g**	2-OH	**47.4 ± 1.8**	881.1 ± 8.0	**18.6**	>100
**2h**	2-OH-5-Cl	137.7 ± 3.0	287.2 ± 12.6	2.1	>100
**2i**	4-CH_3_	91.4 ± 6.2	253.1 ± 9.6	2.8	>100
**2j**	4-CH_3_O	108.5 ± 2.2	279.1 ± 2.9	2.6	>100
**2k**	4-Br	**62.1 ± 0.02**	342.3 ± 11.3	5.5	>100
**2l**	4-CF_3_	**46.8 ± 2.1**	175.0 ± 8.3	3.7	>100
**2m**	4-NO_2_	**60.0 ± 0.4**	190.6 ± 1.1	3.2	>100
**2n**	3-Br	96.9 ± 1.3	166.3 ± 0.8	1.7	ND
**2o**	2-Br	**62.6 ± 3.2**	**54.2 ± 0.1**	0.9	ND
**2p**	3-CF_3_	**49.9 ± 1.4**	**63.6 ± 2.8**	1.3	ND
**2q**	2-CF_3_	**50.0 ± 1.6**	**19.1 ± 0.5**	**0.4**	ND
**2r**	3-NO_2_	81.3 ± 2.6	132.9 ± 10.2	1.6	ND
**2s**	2-NO_2_	**59.7 ± 5.8**	93.1 ± 4.9	1.6	ND
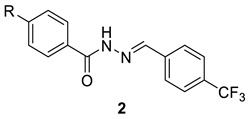					
**2t**	H	**62.9 ± 0.8**	189.8 ± 1.7	3.0	ND
**2u**	CH_3_	75.3 ± 1.5	244.9 ± 7.0	3.3	ND
	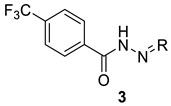				
**3a**	propan-2-ylidene	69.7 ± 5.2	424.3 ± 1.4	6.1	>100
**3b**	cyclopentylidene	69.2 ± 1.0	199.8 ± 13.2	2.9	>100
**3c**	cyclohexylidene	**61.2 ± 1.3**	**74.7 ± 2.7**	1.2	>100
**3d**	1,7,7-trimethylbicyclo-[2.2.1]heptan-2-ylidene	**59.3 ± 1.8**	**57.2 ± 3.4**	1.0	>100
**1** [23]	-	69.4 ± 1.4	204.0 ± 2.7	2.9	>100
Galantamine		1.54 ± 0.02	2.77 ± 0.15	1.8	ND

IC_50_ values are expressed as the mean ± SD (*n* = three independent experiments). ND: not determined. The lowest IC_50_ values for each enzyme are given in bold as well as the most selective inhibitors for both enzymes.

**Table 2:** Blood-brain barrier (BBB) permeability parameters.

Code	M_w_	log *P*	H-Bond Donors	H-Bond Acceptors	tPSA (Å^2^)	Rotatable Bonds	BOILED-Egg
**2a**	292.26	3.66	1	3	41.46	5	✔
**2b**	326.70	4.19	1	3	41.46	5	✔
**2c**	326.70	4.20	1	3	41.46	5	✔
**2d**	326.70	4.37	1	3	41.46	5	✔
**2e**	308.26	3.26	2	4	61.69	5	✔
**2f**	308.26	3.26	2	4	61.69	5	✔
**2g**	308.26	3.43	2	4	61.69	5	✔
**2h**	342.70	4.01	2	4	61.69	5	✔
**2i**	306.28	4.00	1	3	41.46	5	✔
**2j**	322.28	3.67	1	4	50.69	6	✔
**2k**	371.15	4.28	1	3	41.46	5	✔
**2l**	360.25	4.72	1	3	41.46	6	-
**2m**	337.25	3.06	1	5	87.28	6	-
**2n**	371.15	4.29	1	3	41.46	5	✔
**2o**	371.15	4.45	1	3	41.46	5	✔
**2p**	360.25	4.71	1	3	41.46	6	-
**2q**	360.25	4.77	1	3	41.46	6	-
**2r**	337.25	3.05	1	5	87.28	6	-
**2s**	337.25	3.23	1	5	87.28	6	-
**2t**	292.26	3.65	1	3	41.46	5	✔
**2u**	306.28	3.91	1	3	41.46	5	✔
**3a**	244.21	2.93	1	3	41.46	4	✔
**3b**	270.25	3.35	1	3	41.46	4	✔
**3c**	284.28	3.69	1	3	41.46	4	✔
**3d**	338.37	4.60	1	3	41.46	4	✔
**1**	204.15	1.70	2	3	55.12	3	✔
Breakpoint [29]	≤500	≤6.0	<5	<10	≤90	≤5	-

M_w_: molecular weight, tPSA: topological polar surface area.

## Data Availability

Data available on request due to restrictions. The data presented in this study are available on request from the corresponding author. The data are not publicly available due to privacy.

## Supplementary Materials

The following are available online. Table S1: Kinetic parameters for **2l** inhibiting eeAChE calculated from L–B plot; Figure S1: The dependence v_0_/v_i_ vs. concentration of **2a** inhibiting eeAChE; Figure S2: The dependence v_0_/v_i_ vs. concentration of **2g** inhibiting eeAChE; Figure S3: The dependence v_0_/v_i_ vs. concentration of **2k** inhibiting eeAChE; Figure S4: The dependence v_0_/v_i_ vs. concentration of **3c** inhibiting eeAChE; Figure S5: The dependence v_0_/v_i_ vs. concentration of **2d** inhibiting eqBuChE; Figure S6: The dependence v_0_/v_i_ vs. concentration of **3d** inhibiting eqBuChE.

## Sample Availability

Samples of the compounds **1**, **2a**–**2u**, and **3a**–**3d** are available from the authors.
