# Supplementary material for: Hydrazones of 4-(Trifluoromethyl)benzohydrazide as New Inhibitors of Acetyl- and Butyrylcholinesterase
Source: Molecules. 2021 Feb 13;26(4):989. doi: 10.3390/molecules26040989 (PMC7918878; doi:10.3390/molecules26040989)
Supplement: Supplementary file 1 [file molecules-26-00989-s001.pdf]

Supplementary Material

Hydrazones of 4-(Trifluoromethyl)benzohydrazide as New Inhibitors of Acetyl- and Butyrylcholinesterase

Martin Krátký [1,*], Katarína Svrčková [2], Quynh Anh Vu [1], Šárka Štěpánková [2], and Jarmila Vinšová [1]

[1] Department of Organic and Bioorganic Chemistry, Faculty of Pharmacy in Hradec Králové, Charles University, Akademika Heyrovského 1203, 500 05 Hradec Králové, Czech Republic; vuq@faf.cuni.cz (Q.A.V.); vinsova@faf.cuni.cz (J.V.)

[2] Department of Biological and Biochemical Sciences, Faculty of Chemical Technology, University of Pardubice, Studentská 573, 532 10 Pardubice, Czech Republic; katarina.svrckova@upce.cz (K.S.); sarka.stepankova@upce.cz (Š.Š.)

Supplementary Material Content

I. Table S1. Kinetic parameters for **2l** inhibiting eeAChE calculated from L-B plot S2

II. Representative figures (**S1**-**S4**) reporting IC_{50} determination (dependence v_0/v_i *vs.* concentration of selected inhibitors) of selected inhibitors for eeAChE S2-S3

III. Representative figures (**S5** and **S6**) reporting IC_{50} determination (dependence v_0/v_i *vs.* concentration of selected inhibitors) of selected inhibitors for eqBuChE S4

I. Table S1. Kinetic parameters for **2l** inhibiting eeAChE calculated from L-B plot

c(I) μM	K_M (mM)	V_m (s^{-1})
0	0.85	0.32
10	0.69	0.23
20	0.66	0.19
30	0.64	0.16
40	0.64	0.14

II. Representative figures reporting IC_{50} determination (dependence v_0/v_i *vs.* concentration of selected inhibitors) of selected inhibitors for eeAChE

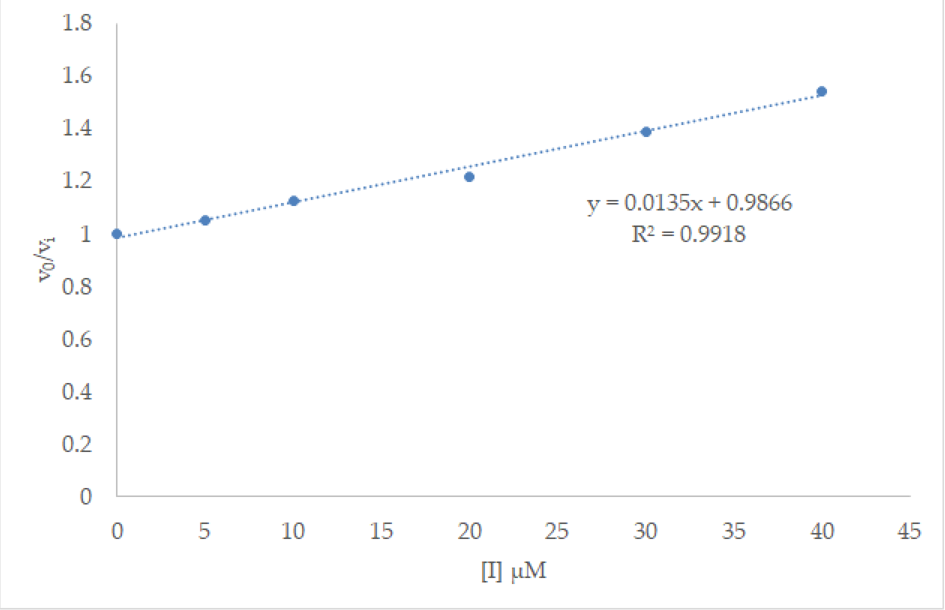

Figure S1. The dependence v_0/v_i *vs.* concentration of **2a** inhibiting eeAChE. Data are the average values from three different experiments.

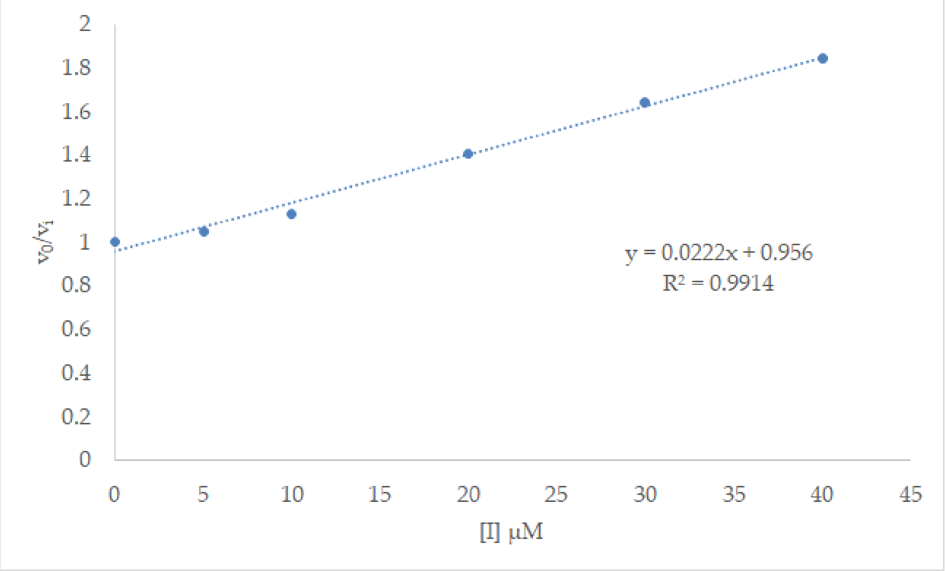

Figure S2. The dependence v_0/v_i *vs.* concentration of **2g** inhibiting eeAChE. Data are the average values from three different experiments.

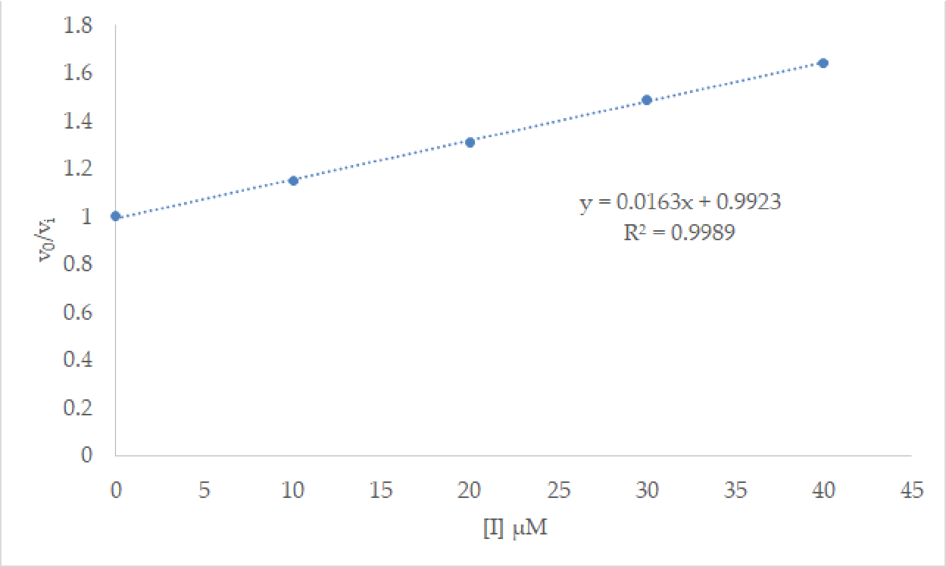

Figure S3. The dependence v_0/v_i *vs.* concentration of **2k** inhibiting eeAChE. Data are the average values from three different experiments.

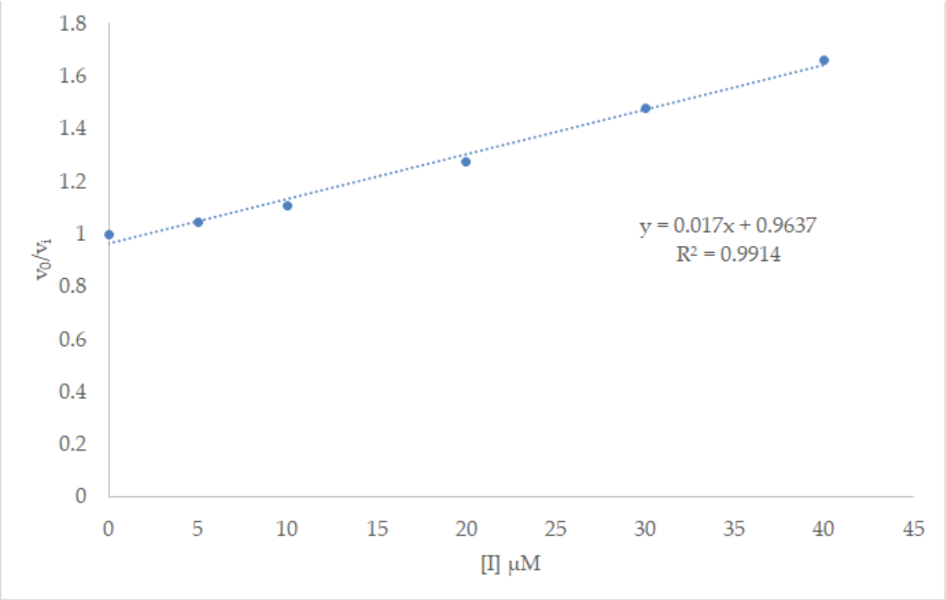

Figure S4. The dependence v_0/v_i *vs.* concentration of **3c** inhibiting eeAChE. Data are the average values from three different experiments.

III. Representative figures reporting IC_{50} determination (dependence v_0/v_i *vs.* concentration of selected inhibitors) of selected inhibitors for eqBuChE

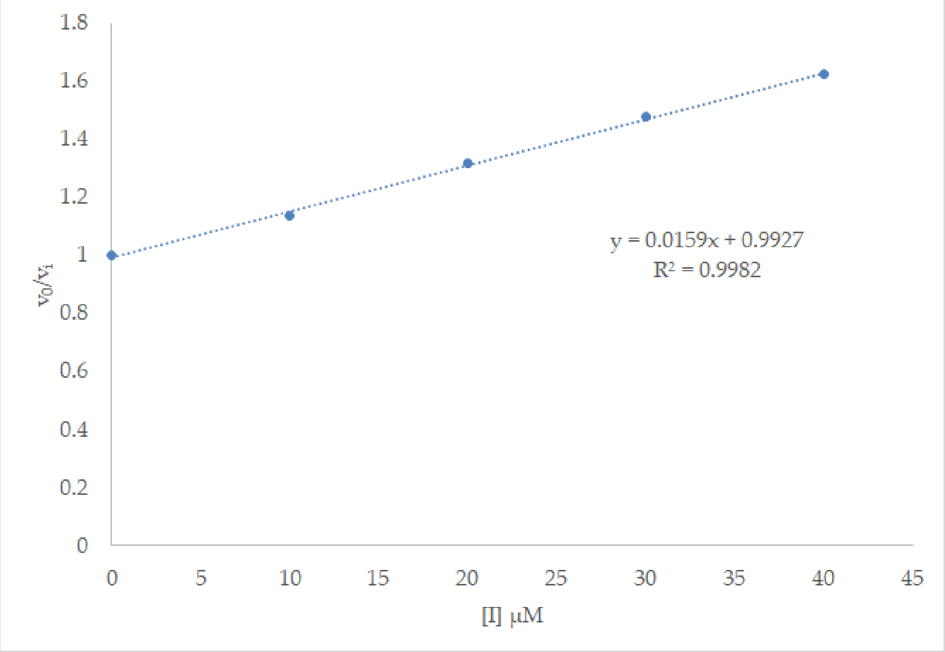

Figure S5. The dependence v_0/v_i *vs.* concentration of **2d** inhibiting eqBuChE. Data are the average values from three different experiments.

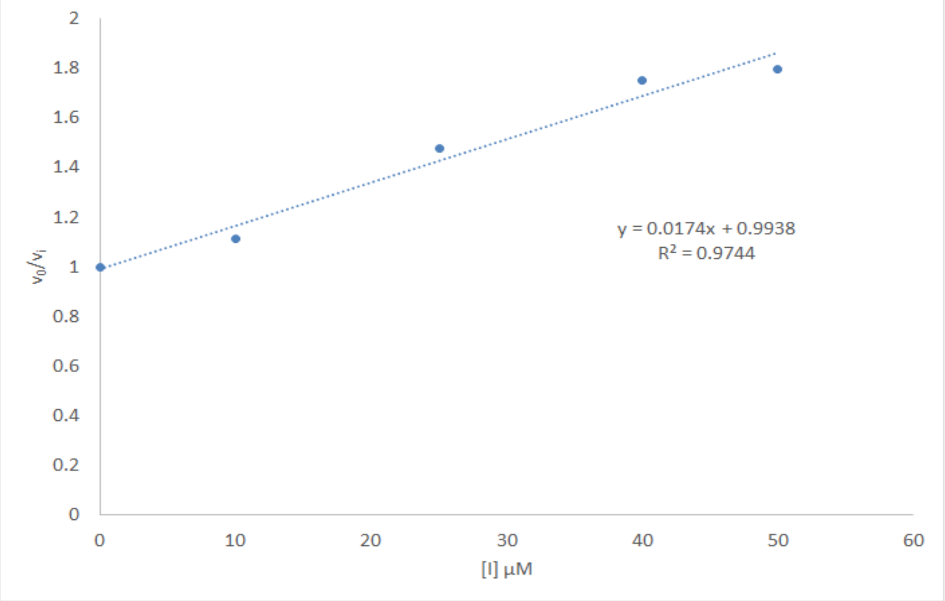

Figure S6. The dependence v_0/v_i *vs.* concentration of **3d** inhibiting eqBuChE. Data are the average values from three different experiments.
